# Retinol Assessment Among Women and Children in Sahelian Mobile Pastoralists

**DOI:** 10.1007/s10393-012-0781-7

**Published:** 2012-07-24

**Authors:** M. Bechir, E. Schelling, K. Kraemer, F. Schweigert, B. Bonfoh, L. Crump, M. Tanner, J. Zinsstag

**Affiliations:** 1 Centre National de Nutrition et de Technologie Alimentaire du Ministère de la Santé Publique au Tchad, BP 440, N’Djamena, Chad; 2 Swiss Tropical and Public Health Institute, The University of Basel, Socinstrasse 57, 4002 Basel, Switzerland; 3 The University of Basel, Petersplatz 1, 4003 Basel, Switzerland; 4 Sight and Life, P.O. Box 2116, 4002 Basel, Switzerland; 5 Institute of Nutritional Science, University of Potsdam, Arthur-Scheunert-Allee 114–116, 14558 Bergholz-Rehbrücke, Germany; 6 Centre Suisse de Recherches Scientifiques en Côte d’Ivoire, P.O. Box 1303, Abidjan 01, Côte d’Ivoire

**Keywords:** vitamin A, retinol, nomadic pastoralist, Chad

## Abstract

Micronutrient deficiencies are widespread in developing countries, particularly in remote communities such as mobile pastoralists. The nutritional and vitamin A status of this population is not well-documented in Chad. This study assessed serum retinol levels among women and children under five-year-old in nomadic and semi-nomadic pastoralist and rural-settled communities, who are similarly exposed to risk factors such as gastrointestinal parasitic infection, anaemia and emaciation. The novel method of portable fluorometry was used for the first time to measure β-carotene and retinol levels in a pastoral nomadic area. Moderate level blood retinol deficiency (<0.7 μmol/L) was observed in 5% (CI 1–11) of nomadic, 29% (CI 13–45) of semi-nomadic and 22% (CI 8–35) of sedentary women. In children, 1% (CI 0.1–4), 17% (CI 9–25) and 28% (CI 18–39), respectively, had moderate level blood retinol deficiency. In nomadic communities, women and children had blood retinol levels close to normal. Deficiency of retinol was strongly linked with lifestyle (nomadic, semi-nomadic and settled) among women and lifestyle and age among children. The results support an ecological linkage between human retinol levels and livestock milk retinol. This study shows the feasibility of portable retinol and β-carotene measurement in human blood as well as human and animal milk under remote field conditions, but the approach requires further validation.

## Introduction

Worldwide, 250 million school children are estimated to be vitamin A deficient, and night blindness affects 5.2 million preschool aged children and 9.8 million pregnant women, (McLaren and Frigg 2002; WHO 2009). The majority of those affected are living in the poorest regions of Africa and Asia (Maida et al. 2008). Vitamin A (VAD) and other micronutrient deficiencies are referred to as ‘hidden hunger’ (McLaren 2008). Along with iron deficiency, VAD ranks among the ten most important risk factors for disease, causing 1.4% of mortality globally (Chacko and Sivan 2008). Estimates indicate that 50% of mortality due to measles in children under five could be prevented through vitamin A supplementation (Benn et al. 2009; Kraemer et al. 2008). The United Nations Children’s Fund (UNICEF) and the World Health Organization (WHO) integrated vitamin A supplementation into the expanded programme of immunization (EPI) in developing countries (United Nations Children’s Fund 1998), and the Programmes against Blindness in Developing Countries registered xerophthalmia as one of five targeted blindness diseases (Traoré et al. 1998).

Remote communities, such as Sahelian mobile pastoralists, have limited access to health services (Wyss et al. 2004; Zinsstag et al. 2005), and their micronutrient status is not well-documented in Chad. One of the few studies done in a Chadian mobile pastoralist population found that the primary source of dietary retinol was milk and 40% of women were deficient (Zinsstag et al. 2002). In the study, blood samples were transported from Chad to Switzerland for high-performance liquid chromatography analysis. Both food consumption and community socio-cultural aspects determine vitamin A levels. Becquey and Martin-Prevel (2010) showed that higher intake of animal products was significantly associated with a lower risk of micronutrient deficiency. In Ethiopia, Demissie et al. (2009) found an association between VAD and age or household characteristics.

Pasture provides the fodder for livestock throughout most of the world. In remote areas, the milk of cows, goats, sheep and camels is an important source of nutrition for the local people. Livestock milk also provides vitamin A, which is vital for human health. The essential precursors of vitamin A (including β-carotene) are synthesized by plants but not by animals or humans. In grassland areas, seasonal rainfall influences the annual growth of vegetation, including the level of β-carotene in fodder, which then determines retinol levels in livestock, milk and, ultimately, human vitamin A status. Earlier study demonstrated that human serum retinol depends significantly on livestock milk retinol in nomadic pastoralists and supported consumption of goat and cow’s milk as an important source of vitamin A (Zinsstag et al. 2002).

In this study, the aim was to extend these assessments to communities with different lifestyles, including nomadic and semi-nomadic pastoralists who depend nearly exclusively on their livestock for nutritional needs, to further document an ecological linkage between human blood retinol and food sources like livestock milk in the southeastern Lake Chad area. In addition, a portable fluorometric device for on-site retinol and β-carotene measurement was tested. This portable measurement method was developed in Germany using cow’s milk (Kawashima et al. 2009) and evaluated for human blood in the USA and Liberia (Craft 2001), where a correlation of portable fluorescence and retinol concentration of 0.9987, in the range of 0.25–3.5 μmol/L, was reported.

The objective of this study was to assess comparative blood and milk retinol level among women and children under five-year-old in nomadic, semi-nomadic (cattle rearing) and rural-settled communities, to determine the association of human blood and milk retinol with livestock milk consumption and to test the practical use of portable fluorometry devices for human and animal samples in a remote area.

## Materials and Methods

A stratified cross-sectional study between mobile pastoralist and rural-settled women and children under five-year-old was done in October 2008, at the end of the rainy season, among Foulbe nomadic and Arabic semi-nomadic communities living near Lake Chad and sedentary communities of Baltram village in the same area. Sedentary communities were composed of Haoussa, Kanembou, Sara and Hadjarai ethnic groups originating from different regions of Chad. Their data were pooled to represent the sedentary population.

### Sampling

**Figure 1:**
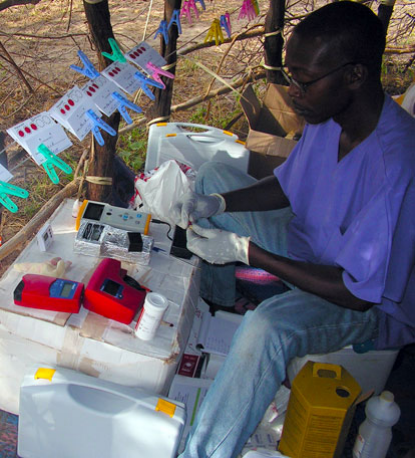
Materials and sample analysis at study site.

The sample size was based on a prevalence of retinol deficiency of 40% among nomadic children (Zinsstag et al. 2002) and 22% among sedentary children, assuming a confidence level of 95%, a power of 90% and a minimum odds ratio of 2.2. Based on these assumptions 150 nomadic/semi-nomadic and 150 sedentary children and their mothers were selected. Random transects were used for the selection of nomadic camps in the study zone, as previously described (Weibel et al. 2008). Pastoral communities were composed of Foulbe pastoralists, who are nomadic throughout the year, and Arabic communities, who practice a semi-nomadic lifestyle with higher mobility during the dry season. Rural-settled women and children were selected in three areas using random numbers from a list of all village areas. The chief of each area and the health centre staff notified mothers of children under age five by megaphone. A mobile laboratory was established in the selected area (Fig. 1).

Milk samples from cattle, sheep and goats were collected from the pooled morning milk of household herds into sterile laboratory tubes. Human milk was collected from volunteer lactating mothers of children from 2 to 22 months of age, by self extraction into sterile laboratory tubes.

### Vitamin A Assessment

Two portable iCheck™ devices (BioAnalyt GmbH, Teltow, Germany) were used to measure retinol and β-carotene in blood and milk (Fig. 1). The iCheck™ Ret 435-1 was used for retinol assessment and the iCheck™ Ret 515-2 for β-carotene. Although rapid methods generally use capillary blood samples (Craft 2001), in this study venous blood was used, as originally developed for veterinary medicine, because of the larger volume of blood required for vitamin A, malaria and haemoglobin (Hb) testing, which included dried blood on filter paper for iron assessment. A syringe was used for venous blood sampling from children.

The same method was used for both devices, with 400 μL blood (or 600 μL of milk) injected into the extraction unit. The contents were shaken for 10 s until the blood changed from reddish to reddish brown. The unit rested for 1 min until separation was complete. The contents of the extraction unit were transferred to the iCheck device, and the value was displayed on the LCD screen. For retinol measurements, the optic unit was enclosed by a black box to prevent infiltration of external light. The same cut-off values were used for blood and serum. Retinol status was considered deficient when concentrations were <0.70 μmol/L, moderately deficient when concentrations were between 0.70 and 0.35 μmol/L and severely deficient for concentrations <0.35 μmol/L. For milk, the retinol activity equivalent (RAE) was calculated as RAE = retinol + 0.167 * β-carotene (FAO and WHO 2001; West and Eilander 2001). The conversion rate of β-carotene from milk is not well known but is likely higher than 6 g because milk has properties of an oil in water emulsion which favours the absorption of β-carotene, compared the vegetables (R. Hurrell, personal communication).

### Anaemia and Intestinal Parasite Status

Hb concentration was measured with the HemoCue Hb301™ photometer (Angelholm, Sweden) after daily calibration with reference blood (130 mg/L ± 1.2). Anaemia was defined as Hb < 120 mg/L for non-pregnant women and <110 mg/L for pregnant women and children under five. Single individual faecal samples were scanned using the Kato-Katz direct observation method for detection of intestinal parasites (Lamy 1980; Golvan and Ambroise-Thomas 1984). Analyses were performed in a mobile laboratory.

### Emaciation

Anthropometric measurements were made using a UNICEF-provided tape board for height and an electronic scale for weight. For children, a *Z*-score was calculated

$$ Z{\text{-score}} = \frac{{{\text{Observed}}\;{\text{value - reference}}\;{\text{value}}}}{{{\text{Standard}}\;{\text{deviation}}\;{\text{of}}\;{\text{the}}\;{\text{reference}}\;{\text{population }}}} $$

Observed values for children were compared with the WHO 2006 growth standards. For women, the body mass index (BMI) was calculated. A *Z*-score weight-for-height index <−2 indicated global acute malnutrition for children and BMI < 18.5 kg/m^2^ indicated malnourishment for women (Bruce 2003).

### Statistical Analysis

Data were double entered into a database (Access 2003^®^, Microsoft, Redmond WA), compared with Epi-Info™ (version 3.5.1, CDC, Atlanta, GA) and statistically analysed (Intercooled Stata^®^ 10, College Station, TX). Different groups were compared by the analysis of variance (ANOVA) for continuous normally distributed variables and by Kruskal–Wallis tests for categorical and ordinal comparisons of small samples of mother’s milk and livestock milk. Logistic regression models related retinol deficiency (<0.7 μmol/L) as an outcome to explanatory variables of anaemia (binary categorisation in anaemic, Hb < 120 mg/L, and non-anaemic, Hb ≥ 120 mg/L), emaciation (binary categorisation in emaciated, *Z*-score < −2, and non-emaciated, *Z*-score ≥−2, for children and in emaciated, BMI < 18.5 kg/m^2^, and non-emaciated, BMI ≥ 18.5 kg/m^2^, for women), multi-parasite infection (binary categorisation in multi-infection and non-multi-infection), age (categorisation in <12, 12–36 and >36 months for children and <25, 25–35 and >35 years for women), lifestyle (categorisation in nomad, semi-nomad and settled) and sex (binary categorisation in male and female for children).

### Ethical Consideration

This study was approved by the Ministry of Heath of Chad (N°236/MSP/SE/SG/DGAS/2007) and by the ethical committee of both cantons of Basel, Switzerland (Ref. Nr. EK: 362/07). In each study site, local health authorities were informed and involved in the study. Collective and individual consent was solicited from heads of camps and village areas and from all participating women. Vitamin A supplements and systemic mebendazole were administered by qualified health personnel.

## Results

Of the 288 collected samples, 260 (90.5%) were analysed and included. The remaining 28 were considered erroneous values and were excluded from statistical analysis.

### Blood Retinol Concentration Among Women and Children

**Figure 2:**
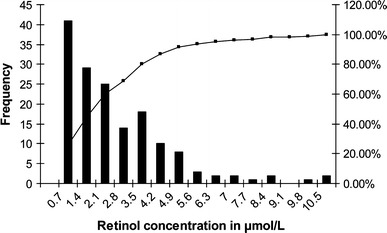
Frequency of blood retinol concentration among children.

Table 1 lists blood retinol levels among women and children of different communities. Nomadic women and children had higher blood retinol concentrations when compared to semi-nomadic and sedentary communities. One in five semi-nomadic and sedentary women and one in twenty nomadic women were moderately retinol deficient. A similar trend was observed in children, with higher levels of retinol deficiency in sedentary children (1 in 4). Figure 2 shows the frequency of blood retinol concentration among children.

**Table 1:** Averages and Proportions of Blood Retinol Among Children and Women According to Lifestyle

	Retinol average			Retinol proportion	
	*n*	Avg. (μmol/L)	95% CI	%<0.7 μmol/L	95% CI
*Women*					
Nomads	54	2.8	2.5–3.2	5	1–11
Semi-nomads	34	2.1	1.4–2.8	29	13–45
Sedentary	14	1.1	0.4–2.1	22	8–35
All	102	2.5	1.8–2.8	17	10–23
*Children*					
Nomads	71	2.8	2.5–3.2	1	0.1–4
Semi-nomads	59	2.1	2.1–2.8	17	9–25
Sedentary	28	0.7	0.3–0.7	28	18–39
All	158	2.1	1.8–2.45	14	10–19
*Boys*					
Nomads	45	2.8	2.5–3.3	0	NA
Semi-nomads	34	2.5	1.4–3.3	10	0.1–18
Sedentary	14	0.4	0.2–0.7	27	12–42
All	93	2.5	1.8–2.8	14	9–20
*Girls*					
Nomads	26	2.8	2.1–3.5	2	0.0–7
Semi-nomads	25	1.8	1.1–2.8	17	4–30
Sedentary	14	0.7	0.2–1.1	27	12–42
All	65	2.1	1.4–2.4	15	8–21

*NA* not applicable.

### Retinol and β-Carotene in Milk

Average retinol values in mother’s milk, the primary food for infants, were slightly higher compared to the blood levels in the same women (Table 2). Mother’s milk retinol content between sedentary and semi-nomadic women was similar, with nomadic women tending towards higher levels of milk retinol (*P* = 0.07). Retinol and β-carotene levels were significantly (two times) higher in livestock milk than in mother’s milk. Sheep milk had the highest retinol levels, followed by goat and cow’s milk.

**Table 2:** Averages of Retinol, β-Carotene and RAEs in Mothers’ and Livestock Milk

	Retinol			β-Carotene		RAEs	
	*n*	Avg. (μmol/L)	95% CI	Avg. (μmol/L)	95% CI	Avg. (μmol/L)	95% CI
*Women*							
Nomads	12	3.6	2.7–4.6	0.07	0.04–0.15	**3.6**	**2.7–4.5**
Semi-nomads	20	2.8	1.8–3.8	0.15	0.06–0.23	**2.8**	**1.8**–**3.8**
Sedentary	5	2.6	0–5.4	0.12	0.09–0.16	**2.7**	**0**–**5.4**
Total	37	3.0	2.4–3.7	0.12	0.08–0.17	**3.1**	**2.4**–**3.7**
*Livestock*							
Cows	25	5.7	5.0–6.6	0.19	0.16–0.22	**5.8**	**5.0**–**6.6**
Goats	19	6.0	4.7–7.4	0.18	0.14–0.19	**6.0**	**4.7**–**7.3**
Sheep	16	6.7	5.6–7.8	0.16	0.12–0.2	**6.8**	**5.7**–**7.9**

RAE = retinol + 0.167 * β-carotene (FAO and WHO 2001).

Bold values indicate the retinol activity equivalent.

**Figures 3–4:**
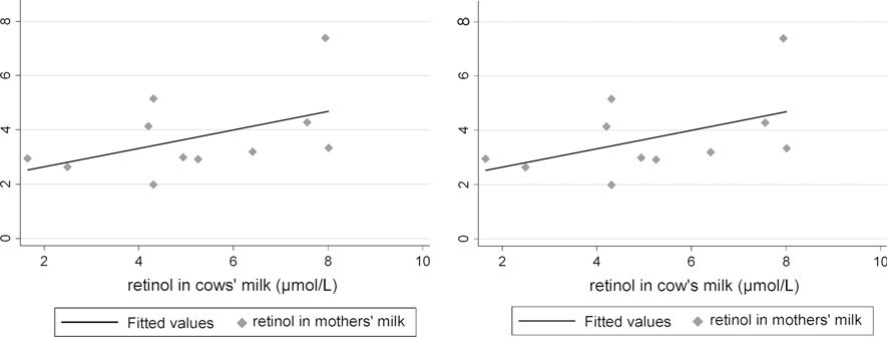
Regression of blood retinol on cows’ milk retinol and regression of mothers’ milk retinol on cows’ milk retinol.

Human blood and mother’s milk retinol values are significantly dependent on cow’s milk retinol levels (Figs. 3–4). Spearman’s rank correlations were positive between cows’ milk retinol and mothers’ blood retinol, where *R* = 0.43 (*n* = 20, *P* = 0.05), between mothers’ blood retinol and mother’s milk retinol, where *R* = 0.24 (*n* = 38, *P* = 0.01), between mothers’ milk retinol and infant blood retinol, where *R* = 0.49 (*n* = 38, *P* = 0.001), and a positive trend between cows’ milk and mothers’ milk, where *R* = 0.48 (*n* = 11, *P* = 0.1). The trend in Figs. 3–4 indicates a linear relationship for these variables within the limits of observation. Average human milk retinol values are slightly higher than those of women serum retinol (Tables 1, 2).

### Risk Factors and Retinol Deficiency

Multivariate logistic regression of the proportion of retinol deficiency in children and women is shown in Table 3. Semi-nomadic and sedentary children have an increased risk compared to nomadic children (OR = 17 and OR = 27; *P* < 0.01, respectively). Likewise, children older than 36 months had significantly higher proportions of retinol deficiency compared to infants <12 months of age (OR = 15; *P* = 0.01). However, there was no significant difference in the risk of low retinol concentration between sex, emaciated and non-emaciated, multi-parasitic infected and non-multi-parasitic infected or anaemic and non-anaemic status in children. Among women, retinol deficiency was only significant in relation to lifestyle. The proportion of retinol deficiency is significantly higher in semi-nomad (OR = 21; *P* < 0.01) and higher, but not significant, in sedentary (OR = 8; *P* = 0.06) compared with nomadic communities.

**Table 3:** Risk Factors for Low Serum Retinol (<0.7 μmol/L) Among Women and Children

	OR	95% CI	*P*
*Children*			
Non-anaemic	1		
Anaemic	0.5	0.2–1.7	0.2
Non-emaciated	1		
Emaciated	0.6	0.2–2.6	0.5
Non-multi-parasitic infection	1		
Multi-parasitic infection	0.6	0.2–2.0	0.4
Age < 12 months	1		
Age 12–36 months	4.1	0.4–14	0.2
Age > 36 months	15	2–38	0.01
Nomads	1		
Semi-nomads	17	3.4–89.7	0.01
Sedentary	27	4.1–131	<0.01
Sex female	1		
Sex male	0.8	0.3–2.5	0.7
*Women*			
Non-anaemic	1		
Anaemic	2.2	0.4–8.2	0.3
Non-emaciated (BMI ≥ 18 kg/m^2^)	1		
Emaciated (BMI < 18 kg/m^2^)	0.3	0.1–1.4	0.1
Non-multi-parasitic infection	1		
Multi-parasitic infection	1.8	0.3–8.6	0.4
Age < 25 years	1		
Age 25–35 years	0.3	0.1–2.0	0.4
Age > 35 years	0.7	0.1–8.5	0.8
Nomads	1		
Semi-nomads	21	2.6–66	0.004
Sedentary	8	1.9–28	0.06

### Retinol Level and Lifestyle

In this study, significant retinol deficiencies were observed in sedentary and semi-nomadic communities. Similar proportions were found in Ghanaian sedentary communities (Lartey 2008). The proportions in those communities were higher than the post-intervention rate of 15–20% (McLaren and Frigg 2001; World Health Organization 1996). The high proportion of retinol deficiency among semi-nomadic communities is likely due to the prevailing livestock management system. During the wet season, semi-nomads remain in a concentrated zone near Lake Chad to sell milk. In contrast, nomadic communities migrate considerable distances, increasing self-consumption of milk as it is not easily marketed during this period. Higher milk consumption likely explains the increased retinol values in nomadic women and children. Consumption of half a litre of milk could contribute 15–20% of the dietary reference intake for vitamin A, as the average reference concentration of retinol in milk is 280 μg/L and the recommended daily intake is 700–900 μg/day (Haug et al. 2007; Insel et al. 2004). High levels of vitamin A were also observed among children of cattle breeders in Mauritania (Borel and Etard 1988). Conversely, higher overall levels (40%) of retinol deficiency were found among nomadic women in Chad (Zinsstag et al. 2002). This could be due to sampling time, which was at the end of the rainy season and during the dry season. In addition, those retinol measurements were done using high-performance liquid chromatography. In the current study, β-carotene values were ten times higher, which may indicate seasonal differences but could also be due to detection method differences.

### Mothers’ Blood/Milk Retinol Level and Infant Blood Retinol Level

Retinol levels in mother’s milk were all close to normal, with the highest values in nomadic women, as observed by Schmeits et al. (1999). Human milk retinol values were slightly higher, an average 0.7 μmol/L, than serum levels. The β-carotene level in mother’s milk was lower than that observed by Bishara et al. (2008). This study showed the interdependencies of vitamin A in infants, who are nourished mainly through mother’s milk. Infant blood retinol was primarily dependent on the mother’s milk retinol. This was also demonstrated by the high proportion of retinol deficiency in children older than 12 months of age, e.g. predominately weaned children, which emphasises the importance of breast feeding and the influence of the maternal diet.

### Ecological Link of Human Blood Retinol Level and Livestock Milk Consumption

Livestock milk was very rich in retinol at the end of the rainy season, and of all ruminant milk evaluated (cow, camel, sheep and goat), sheep milk was richest, which is consistent with findings by the Food and Agriculture Organization of the United Nations (1998), but higher than that recorded by Zinsstag et al. (2002) in the same study area. The relationship between serum retinol level and milk consumption has been previously documented (Murphy et al. 2008), and seasonal variation in nomadic communities likely depends on fluctuation in self-consumption resulting from seasonal variation in marketing practices. The mother’s milk retinol levels followed maternal blood retinol values, which were dependent on the livestock milk retinol content, as this was the main source of vitamin A for the women, confirming earlier observations (Zinsstag et al. 2002). The relationship between animal milk and blood retinol level of consumers and, further, mother’s milk and blood retinol level of children is demonstrated in this study. It could be considered as an ecological link along the food chain from livestock to humans. Further information would be gained through future work examining the link from fodder grass to livestock.

### Retinol and Nutritional Status

In a parallel study, the level of malnutrition in the same study population was investigated, showing a reverse trend with nomadic women experiencing the highest levels of malnutrition compared to semi-nomadic and sedentary women (Bechir et al. 2011). This may indicate that, despite high milk consumption among nomadic women, energy consumption from staple foods, primarily maize and millet is insufficient. According to Wuehler et al. (2011), the nutritional status of young children in the Sahel region would be improved by exclusive breastfeeding for infants, adequate complementary feeding, prevention and/or treatment of malnutrition and the promotion of hygienic practices in environmental sanitation and food preparation and storage. In addition, promotion of local and regional food could be improved. For example, red palm oil, produced in tropical West Africa, is the richest naturally occurring source of β-carotene, and humans can convert this into usable vitamin A as retinol (Rice and Burns 2010; Zagré et al. 2003).

### Limitations of the Study

This study was limited by the small sample size of women’s milk. Therefore, it was not possible to develop further statistical analyses, such as the comparison across age groups of breastfed children.

The study investigated the use of portable devices (Ret 515-2, Ret 435-1 and Hemocue Hb301) in mobile communities. These devices are not designed to operate continuously over long periods of time in harsh environmental conditions, which potentially limits the sample size analysed. In this study, two duplicate devices were utilised to avoid this limitation.

## Conclusion

At the end of the wet season, nomadic pastoralists have higher blood retinol concentration compared to semi-nomadic and sedentary women and children, who seem to be more vulnerable to deficiency. The difference is statistically significant and is likely due to higher milk consumption by nomadic pastoralists, as supported by a positive correlation between blood retinol and livestock milk retinol. However, nomadic pastoralist retinol levels may be decreased during the dry season when milk consumption is reduced as a result of lower production and higher milk sales. In the study area, sufficient milk consumption, complemented by dietary fruits and green vegetables, could maintain adequate retinol status. It is crucial to implement a programme of food education emphasising the importance of animal based sources of vitamin A such as liver and milk, in addition to locally available products such as *Spirulina* or plant based products, despite the limited bioavailability of vitamin A precursors from fruits and vegetables. The results support an ecological linkage between human retinol level and livestock milk retinol. The study shows the feasibility of portable retinol measurement in human blood and human and animal milk under remote field conditions, but it requires further validation.
